# SPOTing Acetyl-Lysine Dependent Interactions

**DOI:** 10.3390/microarrays4030370

**Published:** 2015-08-17

**Authors:** Sarah Picaud, Panagis Filippakopoulos

**Affiliations:** 1 Structural Genomics Consortium, Nuffield Department of Medicine, Oxford University, Old Road Campus Research Building, Roosevelt Drive, Oxford OX3 7DQ, UK; E-Mail: sarah.picaud@sgc.ox.ac.uk; 2 Ludwig Institute for Cancer Research, Nuffield Department of Medicine, Oxford University, Old Road Campus Research Building, Roosevelt Drive, Oxford OX3 7DQ, UK

**Keywords:** lysine acetylation, bromodomain, epigenetic readout, SPOT assay, recognition motif

## Abstract

Post translational modifications have been recognized as chemical signals that create docking sites for evolutionary conserved effector modules, allowing for signal integration within large networks of interactions. Lysine acetylation in particular has attracted attention as a regulatory modification, affecting chromatin structure and linking to transcriptional activation. Advances in peptide array technologies have facilitated the study of acetyl-lysine-containing linear motifs interacting with the evolutionary conserved bromodomain module, which specifically recognizes and binds to acetylated sequences in histones and other proteins. Here we summarize recent work employing SPOT peptide technology to identify acetyl-lysine dependent interactions and document the protocols adapted in our lab, as well as our efforts to characterize such bromodomain-histone interactions. Our results highlight the versatility of SPOT methods and establish an affordable tool for rapid access to potential protein/modified-peptide interactions involving lysine acetylation.

## 1. Introduction

In the post-genomic era, high throughput technologies have entered the biochemistry laboratory allowing for rapid generation of materials that have been used to successfully dissect and interrogate complex biological systems. One of the challenges in chromatin biology has long been the study of interactions governed by small chemical modifications, which are deposited on proteins including histones by “writer” enzymes removed by “eraser” enzymes and interpreted by “readers”, effector protein-interaction modules. Of particular interest are protein:protein interactions that result in “sensing” (or readout) of post translational modifications (PTMs), further resulting in cellular response to the chemical signals encoded by a particular PTM. Lysine acetylation is one of the most abundant PTMs found in cells [1], deposited by acetyl-transferases and removed by de-acetylases, which in the context of chromatin biology and histone proteins are called histone acetyl-transferases (HATs) and histone deacetylases (HDACs). Acetylation of the lysine side-chain results in its neutralization, thus affecting electrostatic interactions between DNA and histones. Weakening of the forces that “glue” the DNA onto histones facilitates the un-wrapping of nucleosome structure, allowing for the transcriptional machinery to locate and dock to accessible loci, that are as a consequence actively transcribed. Recognition of the acetylated lysine residues is primarily initiated by bromodomains (BRDs), evolutionary conserved domains that accommodate the neutralized lysine residue into a small hydrophobic groove within their structure, effectively interpreting the acetylation signal and facilitating the assembly of larger complexes [2]. The precise acetyl-lysine containing linear motifs recognized by bromodomains remain largely unknown, however recombinant bromodomains are readily available [3], allowing for rapid large-scale identification of potential interacting peptides employing available technologies.

Peptide array technology has been established in the early 90s when the SPOT technique was introduced, allowing for rapid parallel synthesis of many peptides on a planar surface, typically cellulose [4]. The technology and its applications have been previously reviewed elsewhere [5,6]. Briefly, Fmoc-protected amino-acids are iteratively added to a functionalized cellulose support resulting in 50–100 nmol of peptide in SPOTs with a diameter of about 8 mm, dispensed in droplets of up to 1 μL. Synthetic yields have improved dramatically over the years and the quality and purity of the resulting peptides is relatively high [7,8]. Peptide arrays have been used to probe protein:protein interactions in the past by providing a versatile platform whereby a target protein can be sectioned into short peptides which are immobilized on a solid support and then a second protein can interact with these peptides in a Western-blot fashion. For example, short sequences within nucleoporins (nuclear pore complex proteins) where shown to bind to the nuclear carrier importin-β employing immobilized short peptides in a SPOT array assay. The use of overlapping peptides allowed to cover the entire sequence of the target protein, thus identifying and mapping individual binding domains [9]. This type of assay can be extended to account for PTMs within a given protein by utilizing modified amino-acids as building blocks, resulting in peptides that carry a variety of post translational modifications. This type of methodology has allowed for the evaluation of antibodies, as well as the identification of linear motifs that contain one or more post translational modifications, leading to novel insight and highlighting the potential of SPOT techniques to rapidly scale and cover large numbers of interacting sequences in the form of short peptides [10]. Many of these modifications are of particular interest in the context of chromatin biology given the rich repertoire of PTMs found on histones [11]. Applications using lysine methylation [12] and acetylation [3], as well as serine/threonine phosphorylation [13], have greatly facilitated our understanding of PTM-contributions to protein:protein interactions. Such applications have also allowed for evaluation of the antibody tools used to detect these modifications in cells. A variation of the SPOT method allows dissolving part of the cellulose support after peptide synthesis resulting in free peptides in solution. This is achieved by utilizing discrete cellulose substrate discs, in what is known as the CelluSpot™ method. Peptides can then be immobilized on glass slides and several copies of each slide can be generated from a single CelluSpot™ synthesis. This method is well documented and can be easily adapted for use in the laboratory [14].

### 1.1. Validation of Commercial Antibodies Using Peptide Arrays

Validation of commercial antibodies targeting histone modifications is one of the applications where high density peptide arrays offer an advantage, as the target peptide sequences can be systematically explored. This is of particular importance in systems where target sequences are similar or where many isoforms of a single protein exist. In addition, introduction of adjacent PTMs near a central epitope can help clarify the selectivity and specificity of these biology reagents, particularly before using them in long end expensive experiments such as chromatin immunoprecipitation followed by deep sequencing (ChIP-seq). For example, while an antibody targeting serine 10 phosphorylation on histone H3 (H3pS10) exhibited high specificity for this mark, an H3pT11 antibody turned out to be non-selective, recognizing several modifications on the entire H3 *N*-terminal portion, including pT32, as well as on H2B peptides carrying several different PTMs [10].

CelluSpot™ arrays have also been used to validate 36 commercial antibodies from various sources aiming to determine their specificity towards histone PTMs. Human histone peptides spanning 20-amino-acid N-terminal fragments of each one of the core histones (H2A, H2B, H3 and H4) carrying combinations of 59 PTMs in a 384-well format using internal repeats, were systematically incubated with antibody for 1 h at room temperature. This study yielded robust and reproducible results, with the obtained binding profiles closely matched even when the synthetic origin of the peptides was different. However, despite the fact that most antibodies bound well to the PTM they had been raised for, several failed, suggesting that SPOT techniques are an excellent tool to validate antibodies before using them in complicated biological experiments [15].

### 1.2. Identification of Acetyl-Histone Peptide Interactions Using Peptide Arrays

The availability of affordable synthetic peptide arrays of high density has stimulated several research projects seeking to identify post-translationally modified linear motifs that are recognized by evolutionary conserved effector modules that act as sensors (or “readers”) for these PTMs. Lack of tools that can be used to deposit these modifications *in vitro* directly onto nucleosomes, has made it impossible to study such motifs in the context of histones. However, peptide arrays have now been successfully used to identify numerous histone-dependent interactions that lead to significant understanding of the underlying biology. The technology itself was shown to be effective for various classes of reader modules, including CHROMO WD-40 and MBT domains [10]. Peptides immobilized on a solid support have also been used to determine potential acetylation-dependent recognition motifs interpreted by bromodomains. Despite the low false negative rate of the method, the false positive rate is much higher requiring the use of orthogonal biophysical methods to confirm binding events. Several studies exploring specific interactions with histone modifications, as well as large scale studies systematically exploring the landscape of histone modifications have been published and will be summarized here, providing a wealth of information suggesting that SPOT techniques can yield robust and reproducible results in identifying acetylation dependent interactions.

Binding of yeast bromodomains to acetylated human histone peptides was tested using peptide arrays. Biotinylated peptides were spotted onto commercial SAM Biotin Capture Membranes and the membranes were incubated with 14 GST-tagged recombinant yeast bromodomains at room temperature. Membranes were immunoblotted with a GST antibody and several acetylated histone peptides were found to bind to these BRD modules, although no orthogonal methods were used to verify these findings [16]. This study established that the technology can be used to rapidly assess binding to several acetyl-lysine modules resulting in numerous potential interactions that can be further validated in order to establish the underlying biological significance of histone-acetyl-lysine recognition. Several BRD-containing proteins have been tested using this technology yielding novel potential interactions.

Binding of acetylated histone sequences to the six BRDs of human polybromo 1 (PB1) was determined by using either cellulose SPOT arrays, or peptide microarrays on silicon slides. Two dimensional (2D) ^1^H-^15^N heteronuclear single quantum correlation (HSQC) NMR spectroscopy was then employed to measure the dissociation constant for the interacting peptides. The interaction of the second bromodomain of PB1 with histone H3 acetylated at lysine 14 (H3K14ac) was measured to be 0.5 mM. An NMR structural model was also determined, suggesting insertion of the acetyl-lysine into the cavity of the bromodomain upon binding [17].

The nucleosome-remodeling factor subunit Bromodomain and PHD finger-containing transcription factor (BPTF or Fetal Alzheimer antigen—FALZ) contains BRD/PHD tandem modules which act together to recognize modifications found on histone tails. The selectivity of the BRD module towards H4K16ac or H4K20ac peptides was established using a SPOT array covering all acetylation sites of human histones, printed on a modified cellulose scaffold. A glutathione S-transferase (GST) construct of the bromodomain module of human BPTF was incubated with an array containing duplicates of 96 modified 15-amino-acids long histone peptides and binding was assessed using a GST antibody. Acetylated H4 peptides were further validated employing in solution isothermal titration calorimetry (ITC) in order to determine thermodynamic binding constants. Intriguingly, this study further demonstrated that the BPTF PHD/BRD tandem modules simultaneously engage two heterotypic trans-histone marks, in the context of full nucleosomes, whereby the PHD module engages histone H3K4me_3_ and the BRD module engages H4K12ac or H4K16ac or H4K20ac resulting in significant selectivity as well as affinity increase [18].

A large scale systematic study of histone modifications was also carried out in order to establish the motifs that are recognized by 33 human bromodomains. Peptides covering single acetylation sites of the four core histones (H2A, H2B, H3 and H4) as well as the linker histone (H1–4) were spotted onto cellulose membranes and were incubated with individual recombinant bromodomains carrying a hexa-histidine affinity tag. After overnight incubation, membranes were washed and probed with an antibody targeting the histidine tag. In addition, arrays covering multiple acetyl-lysine modifications on each peptide were used, screened against the bromo and extra-terminal (BET) subfamily of human bromodomain containing proteins. The effect of adjacent modifications was further examined on histone H3, focusing on a central acetyl-lysine mark flanked by modifications on lysines (acetylation, methylation, dimethylation, trimethylation) as well as serines and threonines (phosphorylation). This systematic study identified 485 acetylation-dependent linear motifs recognized by human bromodomains [3]. Interestingly, the histone acetyl-transferase PCAF as well as BRDs present in nuclear body proteins (such as SP140 and SP140L) exhibited non-specific binding to many peptides found in the arrays studied. Some BRDs did not exhibit any interactions with the tested arrays, suggesting that they may be binding to non-histone proteins. Systematic orthogonal characterization of many interacting peptides using isothermal titration calorimetry established that peptides with dissociation constants of 0.5 mM or lower were successfully identified on the SPOT arrays; however some false negatives were also present. Centering of peptide sequences onto an acetyl-lysine epitope resulted in different affinity to sequences where the epitope was shifted towards the N- or C-terminus, suggesting that steric and positional effects may be present and should be considered when screening large arrays. Some BRDs were also shown to require multiple PTMs in order to interact with a particular peptide, suggesting interplay between signaling pathways. For instance, the BRD of BPTF did not bind to H3K4 or H3K4ac, however it strongly recognized H3pT3K4acK9ac. Similarly, the BRD of WD repeat domain 9 (WDR9) showed strong dependence on pS10 and pT11 in order to recognize H3K18ac. All members of the BET sub-class also exhibited high affinity towards peptides carrying two acetylated lysines. Structural characterization of this interaction established that both acetyl-lysines engage the protein by binding simultaneously within the acetyl-lysine cavity site regardless of the sequences flanking the two acetyl marks. The distance required between two acetyl sites in order to initiate interaction with BET bromodomains was also investigated; this was achieved by using a systematic SPOT array which altered the length as well as the linker type between two acetyl-lysine marks. The results were confirmed employing in-solution thermodynamic measurements, further demonstrating the power and versatility of the peptide array methodology [3].

Combinations of the reader domains found in P300 were tested against a commercial peptide array (Active Motif) covering 384 peptides spanning the human core histones (H2A, H2B, H3 and H4) employing glutathione S-transferase (GST) tagged recombinant domains (BRD/RING/PHD, BRD/PHD or BRD alone). Mainly histone H4 peptides carrying multiple acetylations were found to bind to different constructs containing the BRD module. Interestingly, a systematic pattern for the strongest spots was observed, whereby a two or three-amino-acid spacer separated multiple Kac sequences (Kac(X)_2-3_Kac) [19].

The tandem PHD/BRD modules of TRIM24 were also found to interact with 20-amino-acid long histone H3 peptides carrying K9me*_x_* (*x* = 1, 2, 3) or K9ac or K14ac modifications. Peptides were biotinylated and printed onto a streptavidine coated ArrayIt slide prepared with the BioRad VersArray Compact Microarrayer. The interactions were determined by incubating glutathione *S*-transferase fusion tagged TRIM24 PHD-BRD constructs overnight. Orthogonal methods were used to verify the identified interactions, such as in solution isothermal titration calorimetry as well as pull-downs using the same biotinylated peptides [20].

The PHD/BRD/PWWP triple modules of mouse BS69 (Zinc finger MYND domain-containing protein 11, ZMYND11) were found to bind to histone H3 peptides carrying a K36me_3_ modification using peptide arrays. Probing of a small focused peptide array spanning 84 histone peptides (including peptides found on histones H3, H4, H2A and H2B) with modifications on selected lysine (acetylation/methylation) and arginine (methylation) residues, identified a strong interaction with H3K36me_3_ recognized by the tandem reader BRD/PWWP modules of ZMYND11. The membrane was incubated with glutathione *S*-transferase (GST)–ZMYND11 PHD/BRD/PWWP triple domains and staining with an anti-GST antibody revealed strong interaction with H3K36me_3_ peptides (in triplicate). It is noteworthy that no acetyl-lysine peptides bound to the protein, in agreement with the crystal structure of the BRD/PWWP tandem module where the conserved asparagine found in bromodomains, responsible for histone peptide interactions is replaced with a tyrosine residue (Y192 in mZMYND11), sterically blocking the Kac binding cavity of the module. Intriguingly the protein was further found to preferentially bind to the H3.3 isoform carrying a K36me_3_ modification, resulting in co-localization with H3 and H3.3 on gene bodies, functioning as an unconventional transcriptional co-repressor by modulating RNA polymerase II during elongation [21].

The BRD/PWWP tandem module of human ZMYND11 was also recently profiled against a small histone peptide library printed on a cellulose membrane, covering modifications found on histones H3 and H4. Histone H3 peptides carrying unmodified, methylated (nono-, di- and tri-methylated) or acetylated K36 residues, as well as various combinations of histone H4 peptides with acetylated lysines, were found to bind to the BRD/PWWP modules of ZMYND11. A glutathione S-transferase fusion tag was used on the recombinant proteins for detection carried out employing an anti-GST antibody. Identified interactions were further confirmed employing in solution profiling using a fluorescent polarization assay or western blotting using the full length protein [22].

The Zinc-finger and MYND containing protein 8 (ZMYND8) also contains on its N-terminus three epigenetic reader domains (PHD/BRD/PWWP) which were found to bind to histone marks. A commercial focused peptide array covering 384 peptides (Active Motif) was used to probe binding of a GST-ZMYND8 construct spanning the PHD/BRD modules of the protein. Incubation with the array and detection with anti-GST revealed strong binding to histone H4 marks carrying multiple acetylations (K5ac, K8ac, K12ac, K16ac) or histone H3 peptides carrying K36ac or K36me*_x_* (*x* = 2, 3). Pull-downs with biotin-labeled peptides and the GST-PHD/BRD, as well as endogenous full length protein, confirmed binding to H4 acetylated lysine marks. Interestingly knockdown of the endogenous acetyl-transferase TIP60, responsible for depositing K16ac on histone H4, reduced the protein’s ability to bind to histones, as demonstrated by fluorescent recovery after photobleaching (FRAP) assays in U2OS cells stably expressing GFP-ZMYND8 [23].

Degenerate peptide arrays have been successfully used to define the positional specificity of many interactions (reviewed elsewhere [6]). The positional effect on a central acetyl-lysine residue was systematically probed using cellulose peptide arrays. In these arrays, within each 11 amino acid long peptide bearing a central acetyl-lysine mark, one position contained a fixed amino acid (*i.e.*, as one of the 20 natural amino acids as well as pS, pT, pY, methylated lysine and acetylated lysine) while the remaining positions were degenerate (*i.e.*, containing all possible amino-acids at equimolar amounts). The 14 yeast BRD modules tagged with a GST and a His_6_ (as GST-BRD-His_6_), were incubated with this degenerate histone array. Detection was carried out with a GST antibody revealing preferences for acetyl-lysine recognition in a position- and amino-acid dependent context. Tagged yeast BRDs were then successfully used as affinity reagents to purify from cell extract acetylated histone peptides, acting as pan-specific affinity enrichment reagent, suggesting that BRDs can be engineered in several ways in order to increase their selectivity and affinity towards acetylated sequences, making them good tools for affinity purification-based techniques [24].

Acetylation-dependent interactions successfully identified employing SPOT methods highlight the applicability of the technology and its capacity to generate hypothesis driven research in order to understand the wiring underpinning lysine acetylation signaling networks. In an effort to capture the best experimental conditions necessary for scaling peptide membranes to scan large numbers of linear interacting motifs containing acetylated lysine sequences, we explored published protocols and studied the conditions that give good qualitative results which we then compared to in solution biophysical measurements. Here we summarize our control experiments and highlight step-by step protocols that can be used to systematically study acetylation-dependent interactions employing peptide array technologies to identify interactions of biological significance.

## 2. Experimental Section

### 2.1. Membrane Synthesis

Cellulose-bound peptide arrays were prepared according to standard protocols using a MultiPep-RSi-Spotter (INTAVIS, Köln, Germany) employing Fmoc solid phase peptide synthesis according to the SPOT synthesis method and the manufacturer’s instructions. Peptides were synthesized either on amino-functionalized cellulose membranes (Whatman™ Chromatography paper Grade 1CHR, GE Healthcare Life Sciences #3001-878, Little Chalfont, UK) which were prepared by modifying cellulose paper by introducing Fmoc-β-Alanine as the first spacer residue [21], or on already-derivatized commercially available membranes (Amino-PEG500-UC540 Sheets optimized for use with the MultiPep instruments, INTAVIS). Cellulose-bound peptides were grown on the membranes from their C-terminus by using 0.6 M solutions of Fmoc-amino acid-OPfp (GL Biochem Ltd., Shanghai, China) in *N*-methyl-2-pyrrolidone (NMP, Sigma-Aldrich, Gillingham, UK, #494496) activated with 1-hydroxibenzotriazol monohydrate (HOBt, AGTC Bioproducts, Hessle, UK, #AGHOBT-H2O) and *N*-*N*′-diisopropylcarbodiimide (DIC, Fluka distributed by Sigma-Aldrich, #38370) and spotted on the membrane in 100 nL aliquots per spot using a robotic syringe. Residual amino functions were capped by acetylation between amino-acid block depositions with a 5% solution of acetic anhydride (Fisher Scientific, Loughborough, UK #A/0480/PB08) in NMP. Membranes were then washed several times with ethanol (EtOH) and NMP, before Fmoc groups were cleaved with 20% piperidine in *N*,*N*-dimethylformamide (DMF/Piperidine (80/20), AGTC, #AGDMFPIP). These steps were iteratively repeated for each amino acid within each peptide sequence. Following the last coupling step, the acid-labile protection groups of the amino acid side chains were cleaved by using a mixture of 95% trifluoro-acetic acid (TFA, Sigma-Aldrich, #T6508), 3% tri-isopropyl-silane (ACROS Organics distributed by Fisher Scientific, #214920100) and 2% water for 2 h. Membranes were then washed 4 × 30 s with dichloromethane (CH_2_Cl_2_, ATGC Bioproducts, Hessle, UK, #AGBC7002), 4 × 2 min with NMP and 2 × 2 min with 100% EtOH (absolute ethanol, Sigma-Aldrich, #32221) and were left to dry overnight. In order to ensure that each membrane had been synthesized properly, ultraviolet light (UV, λ = 280 nM) was used to ensure that all SPOTed peptides were present.

### 2.2. Choice of Membrane Material

Cellulose is the most common material used to prepare peptide arrays as initially described in the 90s [4]. In order to avoid “ring spot” (or “corona”) effects, where binding occurs mainly on the rim of the SPOT and not in its center, it has been suggested to reduce peptide density starting from about 10 nmol/cm^2^ and optimizing further [25]. Typically, amino-PEG500-UC540 membranes optimized for use with the MultiPep system have a density of about 400 nmol/cm^2^, as determined by UV quantification after coupling of Fmoc-Alanine. We found that acetyl-lysine carrying peptides synthesized on these membranes tend to saturate the readout signal very quickly, suggesting that large amounts are deposited during synthesis. This proved particularly useful when probing for very weak interactions between acetyl-lysine modified peptides and bromodomains, however strong interactions or interactions that involved binding of multiple domains to one peptide resulted in rapid signal saturation and un-interpretable results. We decided to use in-house generated membranes by directly functionalizing a cellulose surface (regular Whatman™ paper) allowing more flexibility in reducing the loading of each SPOT in our arrays by using different concentrations of Fmoc-β-Alanine during the functionalization of the membrane as previously described [26].

### 2.3. Control of SPOT Density

Commercially available pre-modified cellulose membrane sheets are typically used, however they do not offer the flexibility of in-house cellulose-functionalized membranes, particularly in reducing SPOT density. We functionalized membranes by esterification using Fmoc-β-Alanine and DIC (Fluka, distributed by Sigma-Aldrich, #38370) as previously reported [4,27]. Small (10 × 15 cm) sheets of Whatman™ cellulose (Whatman™ Chromatography paper Grade 1CHR, GE Healthcare Life Sciences, #3001-878) were typically incubated overnight (or at least for 2 h) with 10 mL of a solution comprising 0.64 g of Fmoc-β-Alanine dissolved in NMP, complemented with 374 µL of DIC and 317 µL of *N*-methylimidazole (NMI, Fisher Chemicals, Loughborough, UK, #M/4930/PB05). We found that the solution should cover the entire membrane without any bubbles forming in order to obtain consistency in the downstream array generation. Membranes were washed the following day 3 times with NMP for at least 30 s each, before being incubated for 20 min in fresh NMP.

Washed membranes were Fmoc de-protected by incubation (2 times 5 min each) in 20% Piperidine in DMF, followed by wash with NMP and (4 times for 30 s) then twice with EtOH. At this stage membranes were dried and stored at −20 °C or immediately used. Extra care was taken when handling in-house functionalized membranes as they tend to be much softer than the commercially available pre-functionalized ones. We found that they are also very easy to tear when manipulated with pincers. To avoid ring SPOT effects we found that (at least in the case of acetylation-dependent interactions) using 0.3 g of Fmoc-β-Alanine in 10 mL of solution produced the best results without any significant loss of signal.

### 2.4. Membrane Blocking

In order to avoid background binding of a protein/antibody outside the physical boundary of SPOTed peptides, membranes are typically blocked overnight with an appropriate agent. The choice of blocking agent is also critical for the sensitivity of the antibody used for detection. We have tested different blocking agents, including 5% skimmed milk, 5% bovine serum albumin (BSA, Fisher Scientific, #70955) and 5% alkali-soluble casein (Novagen, distributed by Merck-Millipore, Felyham, UK, #70955). We obtained optimal signal to noise ratio using BSA. It is important to note that BSA is also preferred over milk when detecting phosphorylated proteins, although care must be taken to avoid BSA which contains tyrosine phosphorylations resulting in high background when used with anti-phosphotyrosine antibodies.

### 2.5. Probing Protein:Peptide Interactions

Membranes were pre-wetted by rinsing several times with ethanol followed by 3 × 5 min washes with PBST buffer (3.2 mM Na_2_HPO_4_, 0.5 mM KH_2_PO_4_, 1.3 mM KCl, 135 mM NaCl, 0.1% Tween 20, pH 7.4). They were subsequently blocked with 5% BSA in PBST buffer for 8 h at room temperature in order to reduce non-specific binding. After 2 washes with PBST buffer (5 min each) followed by a single wash with PBS buffer (3.2 mM Na_2_HPO_4_, 0.5 mM KH_2_PO_4_, 1.3 mM KCl, 135 mM NaCl, pH 7.4) for 5 min, His_6_-tagged BRDs were added at a final concentration of 1 µM before an overnight incubation in PBS buffer at 4 °C. Each membrane was washed 3 times in PBST buffer to remove any unbound protein, blocked for 1 h with 5% BSA in PBST buffer, and washed again 3 × 5 min with PBST buffer to remove the excess of BSA. His-tag^®^ Antibody HPR conjugated (Novagen, distributed by Merck-Millipore, #71841) was added in 1% BSA/PBST solution at a dilution of 1:3000. After 1 h incubation, membranes were washed 3 × 20 min in PBST buffer to remove any excess antibody. We found the large number of washing steps necessary in order to avoid low signal to background ratio. All incubation and washing steps were performed using a PMR-30 Compact Fixed-Angle Platform Rocker which was set to 30 oscillations per minute.

### 2.6. Quantification and Visualization

Pierce^®^ ECL Western blotting Substrate (Thermo Scientific, distributed by Fisher Scientific, #32106) was used to reveal the bound antibody and chemiluminescence was detected with an image reader (Fujifilm LAS-4000 ver.2.0, GE Healthcare Life Sciences) typically using an incremental exposure time of 5 min for a total of 80 min (or until saturation was reached, in the case of very strong signal). The resulting SPOT intensities were quantified with the Kodak 1D V.3.6.2 Scientific Imaging System. Two profiles were generated for each SPOT, one covering the outer boundary of each SPOT (large profile) and one smaller (co-centric to the large profile) covering ~50% of the large profile (small profile). Numeric values were imported in Microsoft Excel, profiles were averaged and the intensity was normalized throughout the membrane between 0 and 100. We found that we could identify “corona” effects when the smaller profile intensity was 80%–85% of the large profile intensity, requiring manual adjustment of the data and further experiments. Data were binned using arbitrary derived values for each set of experiments by visual inspection of the membranes resulting in classification of SPOTs as “weak” (typically for normalized intensity values between 5% and 20%), “medium” (typically for normalized intensity values between 20% and 60%) and “strong”(typically for normalized values between 60% and 100%). Visualization was carried out with a simple VBScript within Microsoft Excel taking into account the intensity bins described.

### 2.7. Membrane Stripping

In principle, “stripping” of a membrane from the interacting protein it has been exposed to should be possible, resulting in regeneration of the membrane for subsequent use. It has been suggested that membranes can be regenerated up to 20 times [28], however if the binding to the SPOTed peptides is very strong, stripping may not yield a re-usable membrane. Several stripping protocols have been reported [10,27,29] employing different chemicals as well as different washing steps. In principle membranes are treated with chemicals aiming to denature the bound protein (e.g., β-mercaptoethanol, urea, guanidinium) together with a detergent (SDS or Triton X-100) which helps lift the protein from the membranes. Affinity chromatography matrixes, such as Ni-NTA agarose beads (Qiagen, Manchester, UK) or Talon Metal Affinity Resin (Clontech, Mountain view CA, USA), may help trapping the unbound protein, thus avoiding re-deposition onto the membrane. Subsequent treatment with TFA (ranging from diluted to highly concentrated) for up to 12 h, has been used to regenerate commercially available membranes (which seem to be quite resistant to acidic pH). Lower acid concentrations are required for in-house functionalized membranes which tend to be more prone to hydrolysis and loss of the ester used to attach the peptides on the sheet support. It is important that membranes remain wet following probing of interactions, in order to perform stripping steps. In addition, regeneration should be monitored by repeating the detection step in the absence of any protein sample in order to ensure that the membranes are clean and ready for re-use. We had mixed results with the regeneration of membrane carrying acetylated peptides, sometimes resulting in incomplete removal of the bound proteins. For this reason we typically used new in-house functionalized membranes for every experiment in order to avoid incomplete stripping.

### 2.8. Biolayer Interferometry (BLI)

In order to further assess binding to SPOTed peptides we employed biolayer interferometry against a commercially available set of biotinylated histone peptides (AltaBioSciences, York, UK Histone array, Set 4 Histone Acetyl-Lysine library), covering the same modifications studied in our membrane SPOTs, using the Octet RED384 system (FortéBio, Portsmouth, UK). Experiments were performed at 25 °C in BLI buffer (20 mM HEPES, pH 7.5, 150 mM NaCl and 0.5 mM TCEP) using the FortéBio data acquisition software V.7.1.0.100. Biotinylated peptides were first immobilized onto Super Streptavidin biosensors (SuperStreptavidin (SSA) Dip and Read Biosensors for kinetic #18-0011, FortéBio, Portsmouth, UK), pre-equilibrated in the BLI buffer then quenched in a solution of 5 µM Biotin (baseline equilibration 60 s, peptide loading for 240 s, quenching for 60 s, 1000× rpm shake speed, at 25 °C). The immobilized peptides were subsequently used in association and dissociation measurements performed within a time window of 600 s (base line equilibration 60 s, association for 600 s, dissociation for 600 s, 1000× rpm shake speed, at 25 °C). Interference patterns from peptide-coated biosensors without protein were used as controls. After referencing corrections, the subtracted binding interference data were analyzed using the FortéBio analysis software (FortéBio data analysis software V.7.1.0.38) provided with the instrument following the manufacturer’s protocols.

### 2.9. Experimental Protocol

The precise step-by-step protocol used in our experimental pipeline is given bellow, capturing all important observations that lead to reproducible and good quality SPOT assays in our lab (buffer recipes are also provided at the end of the protocol):

**Day 1: Blocking and hybridization of the membrane (TIMING ~9 h)**

1| Rehydrate the membrane: rinse several times with 100% EtOH then equilibrate 3 × 5 min in PBST (see below).

2| Block the membrane with 10 mL 5% BSA in 1X PBST buffer for at least 8 h at room temperature (use a rocking table).

▲ CRITICAL: Always adjust the amount of solution according to the size of the membrane (and the container used to handle it). The membrane must be covered by the solution on every step.

▲ CRITICAL: Milk can be used for blocking but gives much higher background.

3| Wash the membrane 2 × 5 min in 1X PBST buffer then 1 × 5 min in PBS buffer at room temperature.

4| Add 1 µM (final concentration) of the protein of interest diluted in 10 mL 1X PBS buffer (volume adjustable); leave overnight on a rocking table at 4 °C.

**Day 2: Development and reading (TIMING ~5 h)**

5| Wash the membrane 3 × 5 min with 1X PBST buffer to remove any unbound protein.

6| Block the membrane with 10 mL of 5% BSA in 1X PBST buffer for 1 h at room temperature.

7| Wash the membrane 3 × 5 min with 1X PBST buffer.

8| Add HPR-conjugated His antibody (1:3000 dilution) in 1% BSA PBST buffer and incubate for 1 h at room temperature.

9| Wash the membrane 3 × 15-20 min each in 1X PBST buffer.

▲ CRITICAL: These washes are critical to avoid high background.

10| Place the membrane quickly on drying paper to remove any liquid excess.

▲ CRITICAL: The membrane should not be left to dry completely.

11| Develop using an ECL kit; mix an equal amount of the peroxide solution and the Luminol Enhancer solution (toxic so always wear gloves) and cover the membrane. Let the reaction develop for one minute.

12| Remove the excess of the ECL solution and read the result on a chemiluminescence compatible imaging station (we use the ImageQuant LAS-4000 camera set on chemiluminescence settings) first for 1 min to check that the reaction develops properly, then for 80 min with 5 min increments.

13| Save the resulting image data in the TIF graphic format.

**Day 2: Stripping—Part I (TIMING ~3 h)**

14| Rinse the membrane 3 times in distilled water.

15| Incubate the membrane in the Restore Western Blot Stripping Buffer for 30 min at 37 °C.

16| Wash the membrane 3 × 10 min in distilled water at room temperature.

17| Incubate 2 × 45-60 min in Stripping buffer A (see below) at room temperature.

18| Leave overnight at room temperature in Stripping buffer A with 500 µL of NiNTA beads to trap the released His-tagged protein.

**Day 3: Stripping—Part II (TIMING ~4 h)**

19| Incubate 2 × 30 min in Stripping buffer B (see below) at room temperature.

20| Wash 3 × 10 min in distilled water at room temperature.

21| Wash 3 × 10 min in distilled water at 60 °C.

22| Wash 1 × 10 min in distilled water at room temperature.

23| Wash 1 × 30 min in 90% TFA at room temperature.

▲ **CRITICAL:** TFA is a very strong corrosive acid. Always wear gloves and protective goggles and leave the membrane in a fume hood during the reaction.

24| Wash the membrane 2 × 10 min in distilled water at room temperature.

25| Re-equilibrate the membrane 3 × 10 min in 1× PBST buffer.

■ **PAUSE POINT** If the membrane needs to be used the following day, it can be left overnight into 1X PBST buffer.

■ **PAUSE POINT** If the membrane is no longer needed, it can be stored at −20 °C for several months. In this case, the membrane needs to be dehydrated.

26| Wash the membrane 2 × 10 min in 20% EtOH at room temperature.

27| Wash the membrane 2 × 10 min in 50% EtOH at room temperature.

28| Wash the membrane 2 × 10 min in 95% EtOH at room temperature.

29| Let the membrane dry at room temperature overnight, wrap it in aluminum foil and store at −20 °C in a zip bag.

**Day 4: Quality control of the stripping (TIMING ~5 h)**

30| Repeat steps 6 to 13.

31| If the membrane has been properly stripped (no signal apart from the positive controls), repeat steps 14 to 16, then go to step 25 or 26.

32| If the membrane still displays SPOTs in addition to the positive controls, the stripping process can be repeated (steps 14 to 30), but it is possible that a new membrane will have to be re-synthesized.

**Solution/buffer recipes**

**PBS Buffer (20×, 2L):** Prepare by mixing 320 g of NaCl, 8 g of KCl, 57.6 g of Na_2_HPO_4_, 9.6 g of KH_2_PO_4_ in deionized water for a total volume of 2000 mL. Adjust pH to 7.4 with HCl.

**PBST Buffer (20×, 2L):** Prepare by mixing 320 g of NaCl, 8 g of KCl, 57.6 g of Na_2_HPO_4_, 9.6 g of KH_2_PO_4_, 20 mL of Tween-20 in diionized water for a total volume of 2000 mL. Adjust pH to 7.4 with HCl.

**Stripping buffer A (1×, 1L):** Prepare by mixing 573.18 g of guanidinium HCl salt (6M final concentration) and 10 mL Triton X-100 (1% final concentration) in distilled water for a total volume of 1000 mL.

**Stripping buffer B (1×, 1L):** Prepare by mixing 34.38 g of imidazole (500 mM final concentration), 29.22 g of NaCl (500 mM final concentration) and 20 mL of 1 M TRIS.HCl pH 7.5 (20 mM final concentration) in distilled water for a total volume of 1000 mL.

**Materials needed**

His-tag® Antibody HPR conjugated, Novagen, distributed by Merck-Millipore, #71841.
Pierce® ECL Western blotting Substrate, Thermo Scientific distributed by Fisher Scientific, #32106
Bovine Serum Albumin Heat Shock Reagent grade powder pH7.0, Fisher Chemical, BPE 1600-100.
Restore Western Blot Stripping Buffer, Thermo Scientific, #21059.

## 3. Results and Discussion

We previously employed peptide SPOT arrays to probe all possible acetylation sites found on human histones, as well as the effect of neighboring PTMs (including phosphorylation on serine and threonine residues, acetylation of lysine residues and methylation states (mono-, di- and tri-) of lysines) on the recognition of a central acetyl-lysine epitope by human bromodomain protein modules [3]. Although the technology has been in use for several years we had to adapt our own experimental procedures to the system used in order to probe BRD/acetylated lysine interactions. First, we implemented a protocol for detecting BRDs employing hexa-histidine (His_6_) tagged proteins, as our recombinant platform allowed for rapid scale up of highly pure proteins carrying such an affinity tag. We found His_6_ to be sufficient both for affinity purification of the protein as well as for detection on SPOT membranes. We did note that GST-fused bromodomains are also soluble and can be recombinantly expressed and purified to high yields, but we decided to avoid using the bulky GST tag as it does introduce dimerization as well as steric hindrance in our detection experiment. We then explored the effects of peptide density in the generated arrays, by using well established protocols for density reduction based on mixing Fmoc-β-Alanine with Ac-β-Alanine during the first step of synthesis, effectively reducing the capacity of each SPOT within the array. This was particularly crucial for control peptides (in our case His_8_ peptides) that tend to saturate the antibody readout quickly, resulting in un-interpretable results with SPOT overlap.

**Figure 1:**
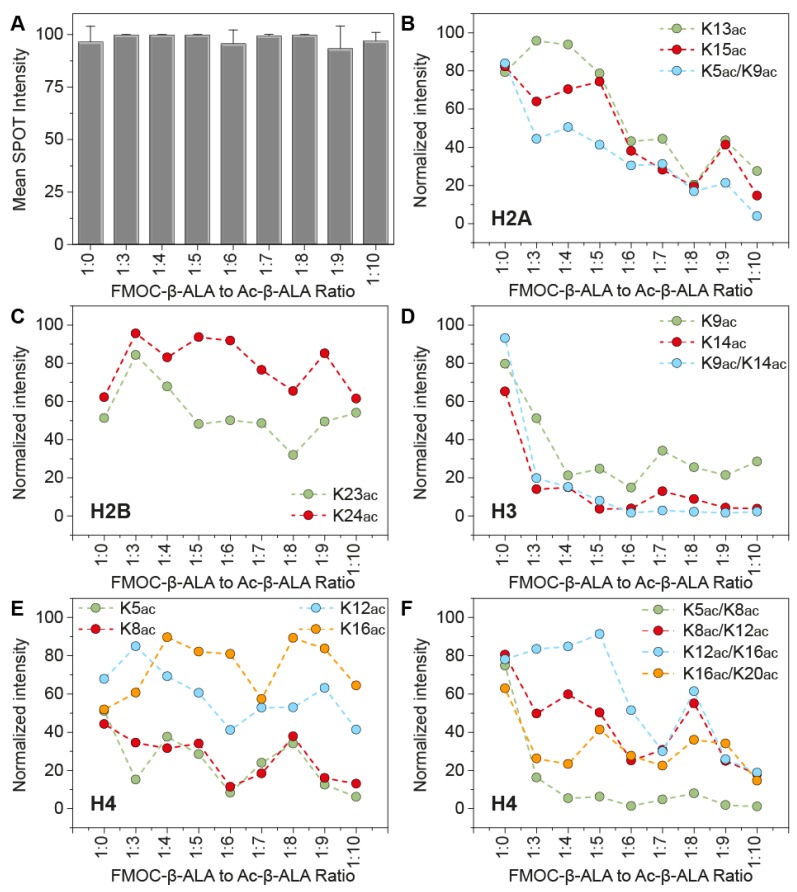
Comparison of SPOT intensity differences following SPOT loading capacity reduction. Peptide arrays were prepared using a ratio of Fmoc-β-Alanine to Ac-β-Alanine that ranged from 1:0 to 1:10 in the first step of synthesis, effectively reducing the amount of peptide found on each SPOT of the array. (**A**) Binding of an His-tag^®^ Antibody HPR conjugated (Novagen) to control peptides carrying 8 histidine residues (His_8_). Reduction of SPOT density did not show a dramatic difference in SPOT intensity, however SPOTs were much smaller and better defined as the SPOT loading was reduced. (**B**–**F**) Normalized SPOT intensity of histone H2A (**B**), H2B (**C**), H3 (**D**) and H4 (**E**,**F**) peptides bound to BRD4(1). Weaker peptides exhibited a drop in intensity as the SPOT density decreased, while non-specific peptides showed large variations. Interestingly, strongly binding peptides carrying 2 acetyl-lysine modifications (e.g., shown in **F**) exhibited overall a decrease in intensity when the SPOT loading was decreased and the binding was 1:1 (as previously determined by in solution binding studies in [3]), however this was not clear when the peptide:protein binding ratio was not 1:1, as in the case of H4K12ac/K16ac.

We synthesized histone peptides that were 15 amino acids long onto cellulose membranes using an INTAVIS MultiPep-RSi-Spotter System. Our design covered previously screened peptides from histones H2A, H2B, H3 and H4, focused on the N-terminal part of the histone sequences and carrying one or more acetylated lysine residues (Table S1). We previously observed that strongly interacting peptides resulted in rapid signal saturation using commercial membranes (Amino-PEG500-UC540 sheets optimized for use with the INTAVIS MultiPep instruments) [3] and therefore attempted to reduce the loading capacity on our arrays to account for this effect. Interestingly, control peptides (His_8_) systematically generated the highest intensity in our detection, however the variability of the control signal within the same array was small (Figure 1A) regardless of the amount of density reduction used during synthesis. We decided that a ratio of Fmoc-β-Alanine to Ac-β-Alanine of 1:6 yielded the best results in terms of control SPOTs (*i.e.*, clear and defined signal with no overlap with adjacent SPOTs) although this is subject to personal preferences rather than numerical justification (Figure S1).

Next, we used the recombinant first bromodomain of bromodomain containing protein 4 (BRD4(1)), which we previously found to strongly interact with two adjacent acetyl-lysine marks and validated its interaction with histone in solution, employing isothermal titration calorimetry as well as by structural biology [3]. We found a large number of peptides carrying one or more acetylated lysine residues on all four histones to interact with BRD4(1). As expected the intensity of the interaction after quantification varied between membranes but there was no clear correlation between SPOT-capacity reduction and binding. Peptides that we previously found to bind in a weak fashion to this protein domain showed a general reduction of SPOT intensity as we reduced the amount of SPOT density (Figure 1B–E). Interestingly, peptides that carried multiple acetyl-lysine marks and were previously found to bind in a 1:1 ratio to BRD4(1) with low double to single digit μM affinity, also exhibited a gradual loss of SPOT intensity as we decreased the SPOT loading (Figure 1F). However several peptides, often carrying a single acetylation site, had non-consistent patterns of SPOT intensity in our entire membrane panel, suggesting that non-specific binding may be taking place (Figure 1C).

**Figure 2:**
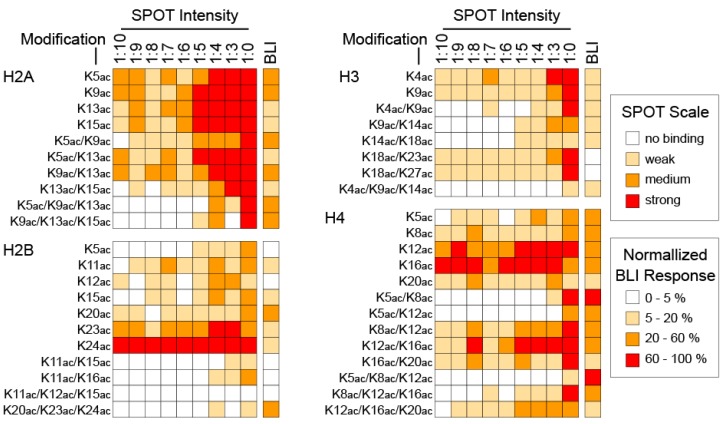
Comparison of BRD4(1) binding to peptides using SPOT arrays and BLI. Membranes carrying histone H2A, H2B, H3 and H4 15-amino acid long peptides with single or multiple lysine acetylation modifications were prepared on cellulose support. Peptide density was controlled using a ratio of Fmoc-β-Alanine to Ac-β-Alanine that ranged from 1:0 to 1:10 in the first step of synthesis, effectively reducing the amount of peptide found on each SPOT of the array. His_6_ tagged BRD4(1) was used to identify binding after incubating membranes overnight at 4 °C. Bound protein was detected using an anti-his antibody (His-tag^®^ Antibody HPR conjugated, Novagen, #71841). SPOT intensity was measured on a luminescent image analyser (Luminescent Image Analyser LAS-4000 Fujifilm) using the KODAC 1D software package (Kodak 1D Scientific Imaging System V.3.6.2.). His_8_ peptides were used as controls for antibody binding and array intensities were normalized between 0 and 100 using the control peptide intensity. The last column on the graphs depicts binding of BRD4(1) to the same peptides derived from a commercial set (AltaBioSciences Histone array, Set 4 Histone Acetyl-Lysine library) carrying biotinylated histone peptides used in a biolayer interferometry experiment (BLI). Binding was normalized for this experiment and the scale is given in the inset. Strongly bound peptides showed binding in both methods while weaker binding peptides were over-represented in the peptide array.

In order to better understand this data we used a commercial peptide array (AltaBiosciences Histone Set 4) which contained biotinylated 20-amino-acid long peptides carrying single or multiple acetyl-lysine modifications on histones H2A, H2B, H3 and H4, with significant overlap with our membrane SPOT design (Table S1). Using biolayer interferometry we measured the ability of recombinant BRD4(1) to bind to these peptides. Comparison of the normalized BLI data to the SPOT results revealed good overlap and explained the non-specific strong signal seen with many peptides on the SPOT assay (Figure 2). For example peptides carrying a K23ac or K24ac mark on H2B systematically showed high intensity profiles by SPOT, however they displayed very weak binding by BLI. Interestingly, the histone H4 peptide K8ac/K12ac which was previously found to bind to two protein modules (both by in solution isothermal titration calorimetry as well as by co-crystallizing with BRD4(1)) exhibited strong binding intensity in both the BLI and SPOT assays and its signal was reduced as a function of SPOT loading, suggesting that a secondary orthogonal assay is necessary in order to introduce confidence in the SPOT results.

Our results highlight the utility of SPOT assays in covering large libraries of potential interacting acetylation motifs for rapid screening against bromodomains. We did not see an obvious advantage of reduced SPOT density for most peptides screened, however SPOT density did affect the intensity of the control peptide signal so we would recommend reducing the loading capacity for control peptides using a 1:6 ratio of Fmoc-β-Alanine to Ac-β-Alanine. Furthermore, using normal Whatman™ paper (Whatman™ Chromatography paper Grade 1CHR, GE Healthcare Life Sciences #3001-878) that we functionalize prior to membrane synthesis we found that most strong interactions were retained when compared to commercial “super-membranes” (data not shown), and we only found small amounts of “corona” effects (which we were able to detect by simple manipulation of intensities in Microsoft Excel, since most “corona” affected SPOTs showed about 80%–85% of the intensity between the two profiles chosen for quantification). Lastly, we used the same home-made membranes to test antibodies capable of recognizing phosphorylated residues (pS, pT and pY) and obtained very clean signal to noise ratios which we used to define the quality of these reagents (Figure S2).

## 4. Conclusions

With the introduction of high throughput technologies in structural and molecular biology a large number of recombinant systems are now available, allowing for parallel testing of protein:protein and protein:peptide interactions. At the same time advances in materials and chemical synthesis have allowed for parallel synthesis of peptides on a small foot-print on solid support. These advances have had a particular impact in chromatin biology, where readers of epigenetic post-translational modifications are now readily available in recombinant form, offering an opportunity to rapidly identify potential interacting linear motifs employing peptide array technologies. The use of enzymes that control the deposition of PTMs on proteins is technologically challenging and as such the reconstitution of nuclesomes in cells, decorated with specific modifications in order to study interactions is a difficult, if not impossible, task. Peptide arrays have paved the way by allowing massive parallel synthesis of peptides carrying PTMs which, in the case of histones, can be recognized by effector reader modules. Lysine acetylation has long being suggested as a potential regulatory modification [2] however the specific linear motifs carrying acetylated lysines that are recognized by reader domains of the bromodomain class have remained elusive, although many known motifs have been disclosed over the years [30]. The use of peptide array technologies has facilitated the identification of various motifs that carry acetylation and are recognized by the bromodomain class of readers as well as the effect of adjacent post translational modifications in the recognition process. Coupled with orthogonal biophysical methods, such as in-solution binding studies and structural biology, peptide arrays offer a powerful tool that can be used to rapidly cover protein space in order to establish acetylation-dependent networks of interactions shedding light into the biological significance of post translational modifications. Further advances in this technology will soon allow for proteome-wide coverage, allowing generating hypothesis for understanding larger networks of interactions governing signaling. The protocols and technology are affordable and mature enough as exemplified by the many reports employing these tools to better understand protein:protein interactions.

## Supplementary Materials

Supplementary materials can be found at http://www.mdpi.com/2076-3905/4/3/370/s1.
