# Supplementary material for: SPOTing Acetyl-Lysine Dependent Interactions
Source: Microarrays (Basel). 2015 Aug 17;4(3):370–88. doi: 10.3390/microarrays4030370 (PMC4996381; doi:10.3390/microarrays4030370)
Supplement: Supplementary file 1 [file microarrays-04-00370-s001.zip › microarrays-91693-Figure S1,S2.docx]

**Supplementary Information**


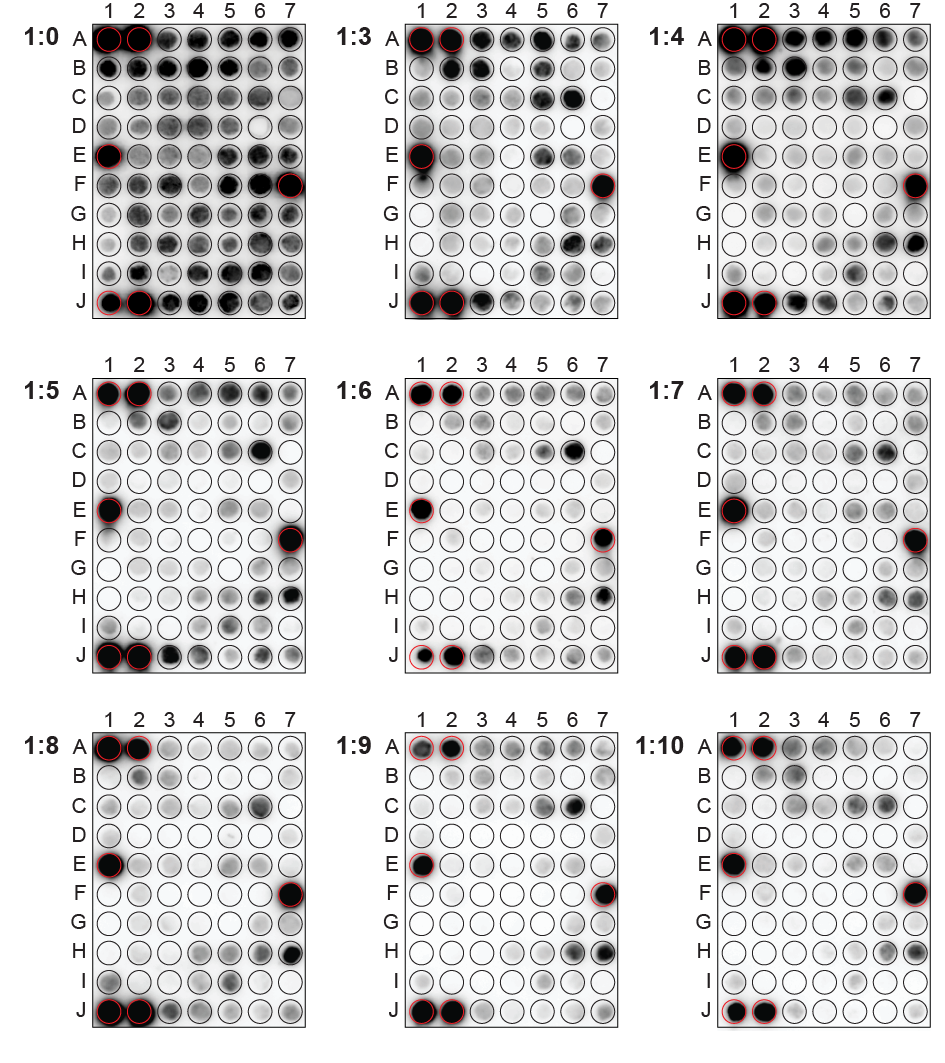


**Figure S1.** Reduction of SPOT peptide loading capacity using Fmoc-β-ALA/Ac-β-ALA mixtures. Membranes were synthesized using the same peptide sequences (Table S1) and an extra initial synthetic step was introduced, whereby a mixture of Fmoc-β-Ala and Ac-β-Ala was deposited on each membrane, accounting for different amounts of peptides found on each array. The arrays were blocked with BSA (for 8 h at room temperature) and were incubated overnight at 4 °C with 1 µM of BRD4(1). After washing, binding was evaluated using an His-tag^®^ Antibody HPR conjugated. Membranes were quantified on a luminescent image analyser (Fujifilm LAS-4000) using the KODAK 1D software (Kodak 1D Scientific Imaging System V.3.6.2.). The Fmoc-β-Ala to Ac-β-Ala ratio is annotated on the left of each membrane. Positive controls (His_8_-peptides) are highlighted with a red circle.


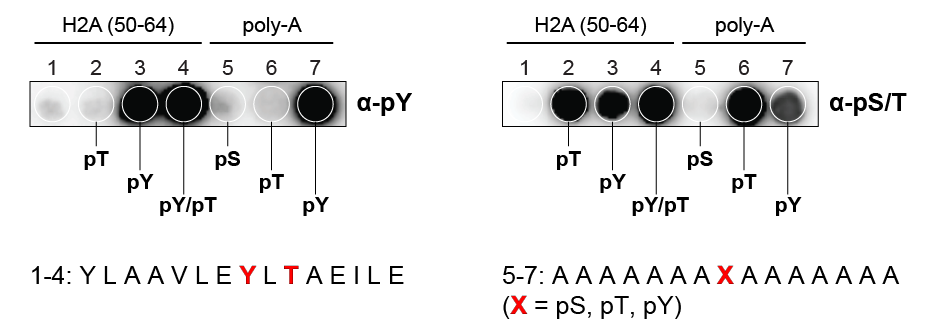


**Figure S2.** Validation of phosphor-tyrosine/serine/threonine antibodies using peptide arrays. A pTyrosine (pY) specific antibody (Anti pY clone 4G10 (Millipore, #05-321) 1:1000 dilution, overnight at 4 °C, secondary antibody: Rabbit to Mouse IgG HPR (Abcam, #ab6728) 1:3000 dilution, 1 h, room temperature) was used to detect pY vs pS and pS sequences histone H2A peptides spanning residues 50–64 and carrying either a pT or pY (or both) modifications as well as three poly-alanine peptides carrying a central phosphorylated epitope (pS or pT or pY). The results (shown on the left part of the figure) demonstrate the specificity of the antibody which only recognized pTyrosine containing sequences. However when the same peptides were tested against a phospho-CK2 targeting antibody (recognizing (pS/pT)DxE sequences; anti-pS/pT (Cell Signaling, #8738S) 1:1000 dilution, overnight at
4 °C, secondary antibody used: goat pAb to Rabbit IgG HPR (Abcam, #ab6721) 1:3000 dilution, 1 h, room temperature), pT and pY sequences were detected but not pS, demonstrating the lack of specificity of this antibody.
